# Switching in Feedforward Control of Grip Force During Tool-Mediated Interaction With Elastic Force Fields

**DOI:** 10.3389/fnbot.2018.00031

**Published:** 2018-06-07

**Authors:** Olivier White, Amir Karniel, Charalambos Papaxanthis, Marie Barbiero, Ilana Nisky

**Affiliations:** ^1^INSERM UMR1093-CAPS, Université Bourgogne Franche-Comté, UFR des Sciences du Sport, Dijon, France; ^2^Acquired Brain Injury Rehabilitation Alliance, School of Health Sciences, University of East Anglia, Norwich, United Kingdom; ^3^Department of Biomedical Engineering, Ben-Gurion University of the Negev, Beer-Sheva, Israel; ^4^Zlotowski Center for Neuroscience, Ben-Gurion University of the Negev, Beer-Sheva, Israel

**Keywords:** phase transition, grip force, internal model, stiffness, uncertainty

## Abstract

Switched systems are common in artificial control systems. Here, we suggest that the brain adopts a switched feedforward control of grip forces during manipulation of objects. We measured how participants modulated grip force when interacting with soft and rigid virtual objects when stiffness varied continuously between trials. We identified a sudden phase transition between two forms of feedforward control that differed in the timing of the synchronization between the anticipated load force and the applied grip force. The switch occurred several trials after a threshold stiffness level in the range 100–200 N/m. These results suggest that in the control of grip force, the brain acts as a switching control system. This opens new research questions as to the nature of the discrete state variables that drive the switching.

## Introduction

A driver switches between different gears, air conditioners switch between on and off states and irrigation mechanisms switch between closed and open circuits. In control theory, hybrid systems are systems with continuous and discrete states. The examples outlined above are switched systems, a subclass of hybrid systems, that are defined as continuous time systems with isolated discrete switching events (Liberzon, 2003). The discrete switching often occurs based on a threshold value of another continuous variable, e.g., the velocity in the former example, the temperature of the thermostat in the second and moisture in the last. Such control systems have many benefits, including economy in control effort (Ben-Itzhak and Karniel, 2008; Karniel, 2011; Leib and Karniel, 2012) and the ability to stabilize otherwise unstable systems (Wicks et al., 1998; Liberzon, 2003; Margaliot and Liberzon, 2006; Lin and Antsaklis, 2009).

Switching is also common in human control of movement. For example, human hand and limb movements are intermittent (Craik, 1947; Navas and Stark, 1968; Neilson et al., 1988; Miall et al., 1993; Doeringer and Hogan, 1998; Squeri et al., 2010; Gawthrop et al., 2014), they switch between different types, such as phase and anti-phase cyclic movements (Kelso, 1984; Levy-Tzedek et al., 2010, 2011) and neural activity states, such as bistability of Purkinje cells firing patterns upon sensory input (Gross et al., 2002; Loewenstein et al., 2005; Yartsev et al., 2009). Several models based on switching were proposed to describe control of standing (Bottaro et al., 2005; Asai et al., 2009; Gawthrop et al., 2014), stick balancing (Gawthrop et al., 2013), and hand movements (Ben-Itzhak and Karniel, 2008; Leib and Karniel, 2012). Intermittent control was proposed to be at least as efficient as continuous control (Loram et al., 2011). Here we present evidence suggesting that the feedforward control of grip force during object manipulation is a switched control system, and we mention several candidate variables that correlate with the switching and that are therefore worth exploring in future investigations.

Many studies have used the modulation of grip force with anticipated load force as an evidence for prediction in the control of voluntary movement (Johansson and Westling, 1984, 1988). Moving an object held in precision grip requires the anticipation of inertial and gravitational forces that may cause its slippage (Flanagan et al., 1993; Flanagan and Wing, 1995). The anticipatory adjustment of grip force generalizes to less usual forms of load force including those dependent on object position (Descoins et al., 2006; Danion and Sarlegna, 2007; Sarlegna et al., 2010; Leib et al., 2015), velocity (Flanagan et al., 2003; Nowak et al., 2004), modified gravitational forces (Augurelle et al., 2003; White, 2015) and when forces are generated by whole body actions such as walking or jumping (Gysin et al., 2008). These predictive mechanisms also generalize to other forms of grip configurations (Flanagan and Tresilian, 1994). Without exception, when load forces are generated by a direct action of the body on the environment, grip force and load force profiles match closely as usually quantified by close-to-zero lags between their peak values, or close-to-zero lags in peak cross-correlation between them.

These studies present evidence for the anticipation of smoothly varying, often self-generated, forces (soft forces). However, in many natural object manipulation tasks, the central nervous system must also adjust grip forces to deal with impulse-like destabilizing forces induced by the nearly instantaneous contact between an object and a hard surface (stiff forces). Several studies also addressed the control of grip force in impact-like tasks: when participants had to anticipate a sudden increase of weight after dropping a ball in a hand-held receptacle (Johansson and Westling, 1988; Bleyenheuft et al., 2009), when opening a drawer to its mechanical stop (Serrien et al., 1999), when hitting an object against a pendulum (Turrell et al., 1999) or a surface (White et al., 2011, 2012) or in a step-down task (Ebner-karestinos et al., 2016). A common observation was the occurrence of a maximum of grip force approximately 60 ms after peak load force that signed the impact. A natural question occurred as to whether this delayed grip force peak resulted from a feedback process. Recently, by studying grip force in catch trials, where load forces are not applied, experiments unambiguously demonstrated this behavior reflects a feedforward process and is not a mere reflex response to a perturbation signal (Bleyenheuft et al., 2009; White et al., 2011). Nonetheless, this feedforward strategy contrasts sharply with the zero-delay coupling observed between grip and load forces when the latter vary smoothly. To sum up, past investigations showed that grip force control in soft and stiff elastic force fields exhibits different feedforward control strategies. This is surprising since the underlying mechanics is described by a single stiffness parameter (k) that varies continuously.

Here, we set out to explore the nature of the transition between these two different feedforward control strategies. We studied grip force adjustment during repeated interactions with virtual objects rendered as elastic force fields. In the repeated interactions, the objects properties varied between soft objects to rigid surfaces or vice versa, resulting in systematically changing impact forces, either increasing or decreasing. We hypothesized that if participants adopt a continuous control strategy, when the stiffness will increase (or decrease) continuously over trials, the grip force—load force delay will continuously increase (or decrease) with respect to the impact. Alternatively, if participants adopt a switching control strategy, we expect to find a stiffness level around which there will be a phase transition in the synchronization between the modulation of grip force and the anticipated load force.

## Materials and Methods

### Participants

Eighteen right-handed adults (14 females and 4 males, 20–40 years old, mean = 24.3, SD = 10.2 years) participated voluntarily in the experiment. All participants were healthy, without neuromuscular disease and with normal or corrected to normal vision. The experimental protocol was carried out in accordance with the Declaration of Helsinki (1964), the procedures were approved by the local ethics committee of Université de Bourgogne and a written informed consent was obtained from all participants. All participants were naïve as to the purpose of the experiments and were debriefed after the experimental session.

### Apparatus and Stimuli

**Figure 1:**
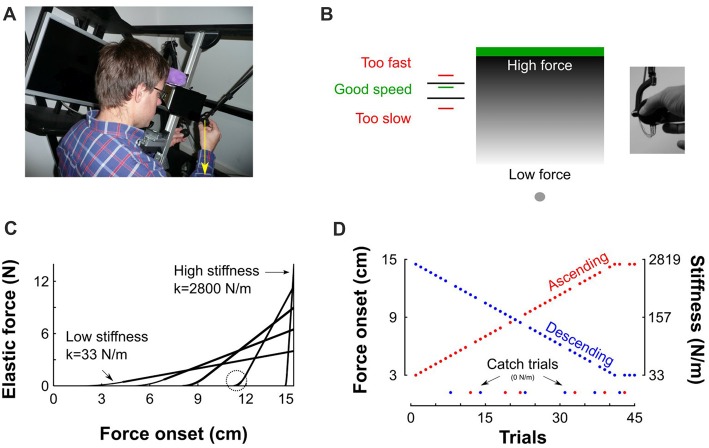
Description of experimental setup and procedures. **(A)** View of the virtual reality stereoscopic display. A participant is seated and holds the transducer with his right hand. The vertical yellow arrow that points downward represents the pushing force operated by the robotic device. **(B)** A gray sphere was moved toward a green target through a parameterized elastic force field. The gray gradient represents the magnitude of the force field for a given stiffness. It is low far from the target (bright gray) and increases when vertical position approaches the target (dark gray). Left: feedback of achieved peak velocity. The right inset depicts the force sensor attached to the end of the robot handle held in precision grip. **(C)** Examples of force-position trajectories of five elastic force fields parametrized by five pairs of stiffness levels and force onset position. Force onset occurred between 3 cm and 14.5 cm above home position. The steeper the slope the stiffer the force field. The circle highlights the second order interpolation between a null force field and a linear elastic force field. **(D)** Structure of “Ascending” and “Descending” blocks, where stiffness increases (red) and decreases (blue), respectively. Catch trials, for which the stiffness is set to 0 N/m, are positioned at the bottom of the trace.

Participants sat in front of a virtual haptic environment with their head on a chin rest (Figure 1A). A mini40 force-torque sensor (ATI Industrial Automation, NC, USA) was mounted on the handle of a robotic device (Phantom 3.0, Sensable Technologies, RI, USA) to record grip force which is the normal force applied by the thumb and the index finger on the transducer (−Fz) and load force $\left(\sqrt{F_{x}^{2}+F_{y}^{2}}\right)$. The 3d positions and forces of the robotic arm were controlled in closed loop at 1 kHz. Participants looked into two mirrors that were mounted at 90 degrees to each other, such that they viewed one LCD screen with the right eye and one LCD screen with the left eye. This stereo display was calibrated such that the physical location of the robotic arm was consistent with the visual disparity information.

### Experimental Procedure

Participants grasped the force sensor with a precision grip (thumb on one side and index finger on the other side, Figure 1B inset). To initiate a trial, participants moved their right hand, displayed as a gray 0.5-cm sphere, into another gray starting sphere (1 cm diameter), displayed at body midline and at chest height. Then, a green target rectangle (12 cm width, 1 cm height) appeared 15 cm above home position (Figure 1B). Participants were instructed to move the cursor straight upward to touch the target and bring it immediately back to home position without stopping at the reversal point. No instructions were provided regarding how they had to adjust grip force. To avoid large trial-to-trial variability in movement kinematics, after each trial, a line was displayed at a height proportional to peak velocity together with lower (45 cm/s) and upper (55 cm/s) bounds displayed as black horizontal segments. The color of the line was red if peak velocity was outside the interval or green in successful trials (Figure 1A). Participants adjusted their movement such that peak velocity fell in that interval.

The target was located inside an elastic force field *F* that was haptically rendered according to $F=\{\begin{array}{cc} 0, & y(t)<y_{0}\\ k\;(y(t)-y_{0}), & y(t)\geq y_{0} \end{array}$, where *y*_0_ is the boundary of the object and k the stiffness value. Such force field emulates a one-sided spring-like object that only resists compression. The more the cursor approached the target, the more effort was required to move it to the target (see gray gradient in Figure 1B). The stiffness of the force field and its onset were varied systematically such that force fields with higher levels of stiffness also onset further along the movement progression (as depicted in Figure 1C for five different force fields). The weakest elastic force field was generated when force onset occurred at 3 cm and linearly ramped up to a maximum force of 4 N along the 12 remaining cm (Figure 1C, *k* = 4/0.12 = 33 N/m). Similarly, the strongest force field was obtained when force onset occurred 0.5 cm below the target’s lower surface and ramped up to a maximum force of 14 N (Figure 1C, *k* = 14/0.005 = 2800 N/m). Force onset (*y*_0_) and stiffness (*k*) pairs, were parameterized independently trial by trial. The transitions between zero force outside of the elastic force field and non-zero force was smoothed with a second order polynomial interpolation (Figure 1C, circle) to avoid mechanical vibrations and overheating of the robot motors, particularly in stiff trials. Consequently, movements were felt as natural and continuous.

The recording session consisted of 10 blocks with 45 movements in each block. In the first five blocks, the stiffness of the elastic force field increased during 41 trials and plateaued for the last four trials (Figure 1D, red “Ascending” blocks). Force onsets were linearly spaced between 3 cm and 14.5 cm by steps of exactly 0.2875 cm. In the last five blocks, force field stiffness decreased over trials (Figure 1D, blue “Descending” blocks). Ten participants started the experiment with the “Ascending” blocks and eight participants started the experiment with the “Descending” blocks. In every block, six trials (13%) were randomly chosen to be catch trials in which the stiffness and onset of the elastic force field were set to zero, effectively vanishing the force field (Figure 1D, green disks). Their order was counterbalanced between participants. In the remaining 39 trials, the natural dynamics remained intact.

### Data Processing and Statistical Analyses

Position and grip forces were recorded at 500 Hz. Grip force rate, velocity and acceleration were obtained using a central-difference algorithm and smoothed with a zero phase-lag autoregressive filter (cutoff 20 Hz). All trials were aligned to movement onset, defined as the time when velocity went above 3 cm/s during at least 100 ms. We also recorded temporal occurrences and values of peak acceleration, grip forces and vertical force field (minmax function in matlab and visual check). The last one corresponded to the time of impact. Finally, we extracted the value of grip force rate at expected force onset. We programmed the trial sequences in such a way to record what stiffness would have been generated in catch trials. Therefore, we know the theoretical stiffness and the corresponding force profile (that is not rendered in catch trials). This measure provides an estimate of feedforward mechanisms of grip force and allows direct comparison between normal and catch trials. To compare real and catch trials, we grouped trials of the same block in seven mini blocks of five trials each (except the first and last mini blocks with 10 trials each). That way, every mini block had both real and catch trials. Because stiffness spanned multiple orders of magnitudes, we sometimes used logarithmic scale to plot these values.

We verified that starting with five “Ascending” blocks (*N* = 10) or five “Descending” blocks (*N* = 8) did not influence any of the above variables (all *F*_(1,17)_ < 1.2, *p* > 0.312). We therefore pooled these two groups together. Quantile-quantile plots were used to assess normality of the data. A three-way ANOVA was conducted on the above variables to assess the effects of stiffness (Mini block, 1–7), Block condition (“Ascending” vs. “Descending”) and Type of trial (“Real” vs. “Catch”). Paired *t*-tests of individual participant means or bootstrap procedures were used to investigate differences between conditions on the above variables. Significance level was set to alpha = 0.05. Data processing and statistical analyses were done using Matlab (The Mathworks, Chicago, IL, USA). Linear fits were calculated with the polyfit function. Partial eta-squared values are reported for significant results to provide indication on effect sizes.

## Results

Participants grasped a force transducer attached to the handle of a haptic device and produced vertical arm movements to touch a virtual target situated 15 cm above home position (Figure 1A). The robotic device generated a resistive vertical elastic force field that was parameterized by the stiffness of the field. As trials progressed, the stiffness of the force field either increased or decreased between two extremes, and force onset was shifted further from or closer to movement onset, depending on block condition (“Ascending” and “Descending”, respectively, Figure 1C). Force fields with the lowest stiffness were similar to a soft elastic force fields that are typically used in other studies (Descoins et al., 2006). In contrast, the force fields with the highest stiffness resembled collisions between the hand-held device and a rigid surface (White et al., 2011, 2012). To measure the feedforward grip force adjustment, we interspersed catch trials, in which visual information was available but no forces were applied. We explored the transition in grip force control between these two extremes.

### Grip Force Is Different When Interacting With High-Impact and Low-Impact Elastic Force Fields

**Figure 2:**
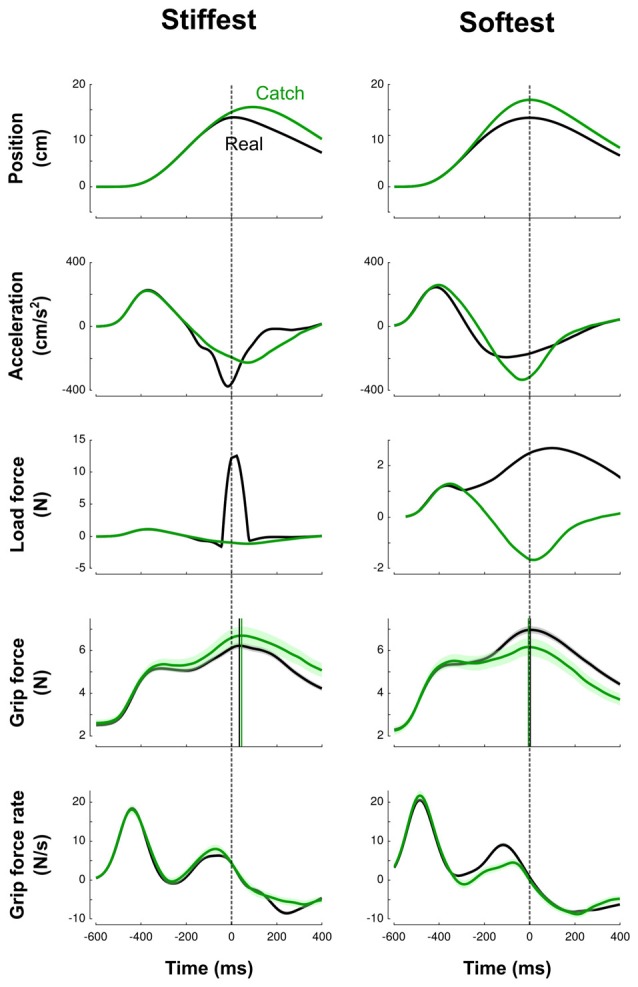
Averaged traces corresponding to the stiffest (left column) trials and softest (right column) trials across blocks and participants. Top to bottom: vertical position, vertical acceleration, load force, grip force and grip force rate are depicted as a function of time. Black and green lines correspond to real and catch trials, respectively. All traces are aligned with peak of impact (vertical dashed cursor across panels, time 0). Cursors for the grip force traces are positioned at their respective maximum. The lags calculated between peaks of grip and elastic forces illustrate the difference between high-stiffness (lag = 40 ms, SD = 6 ms) and low-stiffness (lag = 4 ms, SD = 6 ms) conditions. Error shade areas correspond to SEM. Traces are not normalized.

Figure 2 illustrates trials in the stiffest condition (left column, solid line) and in the softest condition (right column, solid line) averaged across blocks and participants in a force field trial (black line) and in a catch trial (green line). This figure highlights how the trials differ between the two extreme conditions. In the high-stiffness condition, the vertical position increased until the target was touched at 15 cm and then decreased to return to the home position. Participants achieved mean peak velocities of 49.9 cm/s (SD = 8.3 cm/s), within the prescribed 45–55 cm/s interval. The elastic force field was null until the position of the hand reached the boundary of the field at *x* = 14.5 cm and then increased up to 13.75 N (middle row, black line). The vertical pushing force increased when the cursor approached the target and decreased on its way back to the starting position. Grip force increased first to counteract the inertial force (Figure 2, Acceleration row) induced by accelerating the mass of the device and exhibited a first local peak synchronized with a local peak in the load force. Then, after a small dip, grip force increased again in anticipation of the contact. This is also reflected by positive grip force rates for 200 ms before impact (bottom row). However, in this stiffness condition, peak grip force was clearly delayed by 40 ms (SD = 6 ms) after the peak of the elastic force field.

In the low-stiffness trials (Figure 2, right column), the position and acceleration trajectories resembled those for the high-stiffness trials. However, the elastic force field was smoother: it increased for 400 ms up and reached a 4-N peak. Grip force and grip force rates paralleled the traces observed in the stiff condition with one notable difference: peak grip forces were synchronized with the impact, both in real and catch trials (mean = 4 ms, SD = 6 ms).

### Motor Planning Is Similar Between Real and Catch Trials

It is important to stress that the delay between grip force peak and load force peak observed in high-stiffness trials is not a consequence of a feedback control, but rather a feedforward control that includes a delay. We observed the same behavior in catch trials without the presence of the resistive elastic forces (Figure 2, green lines). The green vertical line is positioned at the time when peak elastic force would have occurred given the vertical position. In particular, grip force peaks were delayed by a similar amount relative to impact or expected impact in both real and catch trials.

Due to the absence of resistive forces, different kinematic profiles after *t* = 0 (the anticipated onset of the perturbation) were induced, and the peak position overshot the target. Moreover, in these trials, participants expected a force ramp but did not feel it. It could be argued that these errors signals could have driven a feedback adjustment of grip force rather than reflect a feedforward strategy even in catch trials. We conducted complementary analyses to show that several parameters characterizing motor planning were the same between real and catch trials.

First, we extracted the value of grip force rate, a reliable index of feedforward grip force control (White et al., 2008), at the time of expected force rise. A *t*-test failed to report a difference between real and catch trials on grip force rate (*t*_17_ = 0.5, *p* = 0.642).

Second, we examined the trial-by-trial variations, and verified that grip force rate in catch trials were not statistically different from grip force rate in the real trial that immediately preceded or succeeded them. To do so, we defined two additional variables by subtracting grip force rate in the previous (*R*_t-1_) or next real trial (*R*_t+1_) from grip force rate in the catch trial (*C*_t_) between them (*C*_t_ − *R*_t-1_ and *C*_t_ − *R*_t+1_). The ANOVA reported no difference for *C*_t_ − R_t-1_ (Mini block: *F*_(6,238)_ = 1.8, *p* = 0.109; Block condition: *F*_(1,238)_ = 0.7, *p* = 0.414) and for *C*_t_−R_t+1_ (Mini block: *F*_(6,238)_ = 0.5, *p* = 0.821; Block condition: *F*_(1,238)_ = 1.0, *p* = 0.329).

Figure 2 shows that acceleration traces diverge around the perturbation induced by the elastic force field. Acceleration signals also reflect the output of the motor plan. We conducted a last analysis to quantify how acceleration profiles differed between catch and real trials. To do so, we considered mini blocks because they included real and catch trials. We averaged acceleration traces in real trials and in catch trials separately, per participant and per mini block. Then, we ran an independent iterative *t*-test that compared values of acceleration between both trial types, from trial onset to maximum elastic force (time = 0), and by 20-ms bins. This allowed us to extract the exact time point from which both acceleration traces diverged significantly for at least 150 ms (*p* < 0.05). We identified a divergent point unambiguously on every averaged acceleration profile in mini blocks (all *t*_17_ > 4.1, all *p* < 0.001, all $\eta_{\text{p}}^{2}$ ≥ 0.56). Acceleration values between catch and real trials exhibited a divergent point some 30 ms after force onset in every stiffness condition. This analysis clearly shows that motor planning, in terms of its consequences measured through acceleration, is not affected by trial type.

Based on these analyses, we conclude that in both trial types: (1) motor planning was similar; and (2) grip forces is adjusted on a feedforward manner.

### Grip Force Switches Between Different Control Strategies

**Figure 3:**
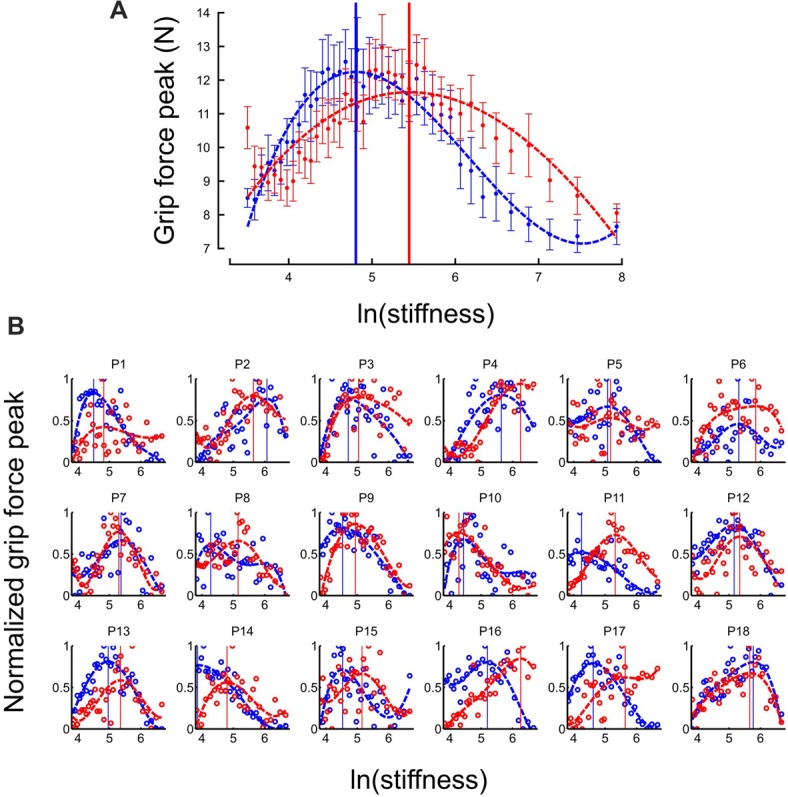
Grip force is adapted to stiffness. **(A)** Distribution of grip force maximum (averaged across participants) in function of ln(stiffness) in “Ascending” (red trace) and “Descending” block conditions (blue trace). The dashed lines are the best fourth order polynomial fits of each series. Goodness of fit: *r*^2^ = 0.72 in “Ascending” condition and *r*^2^ = 0.84 in “Descending” condition. The vertical cursors are positioned at peak grip force (“Ascending”: 5.07; “Descending”: 4.89, which correspond to stiffness of 159.7 N/m and 133.2 N/m, respectively). Error bars are between participants SE. **(B)** Individual normalized plots showing the same behavior at the participant level (except for participant 14 in the “Descending” condition).

At the individual trial level, grip force always exhibited a clear peak over time (see Figure 2). Interestingly, the distribution of grip force peaks *themselves* as a function of stiffness of the elastic force field also reached a global extremum, both in “Ascending” and “Descending” blocks (Figure 3A, average across participants). This observation also held in individual participants except for participant 14 in the “Descending” block condition (Figure 3B). To compare these global grip force extrema as a function of stiffness, we fitted polynomial models to the data averaged across participants (Figure 3A, dashed lines) or for each participant (Figure 3B, dashed lines). Since inter-participant variability was large, we adopted a bootstrap method to test whether grip force peaks of participant fits occurred at the same stiffness level between “Descending” and “Ascending” block conditions (sample = 18, repetitions = 10,000, SD = 0.18). The 95%-confidence interval (CI) of the difference between both population means was 0.08–0.77, excluding zero (at *p* = 0.014). Hence, we conclude that the extremum for the “Descending” block condition occurred at lower stiffness values than in the “Ascending” block condition.

**Figure 4:**
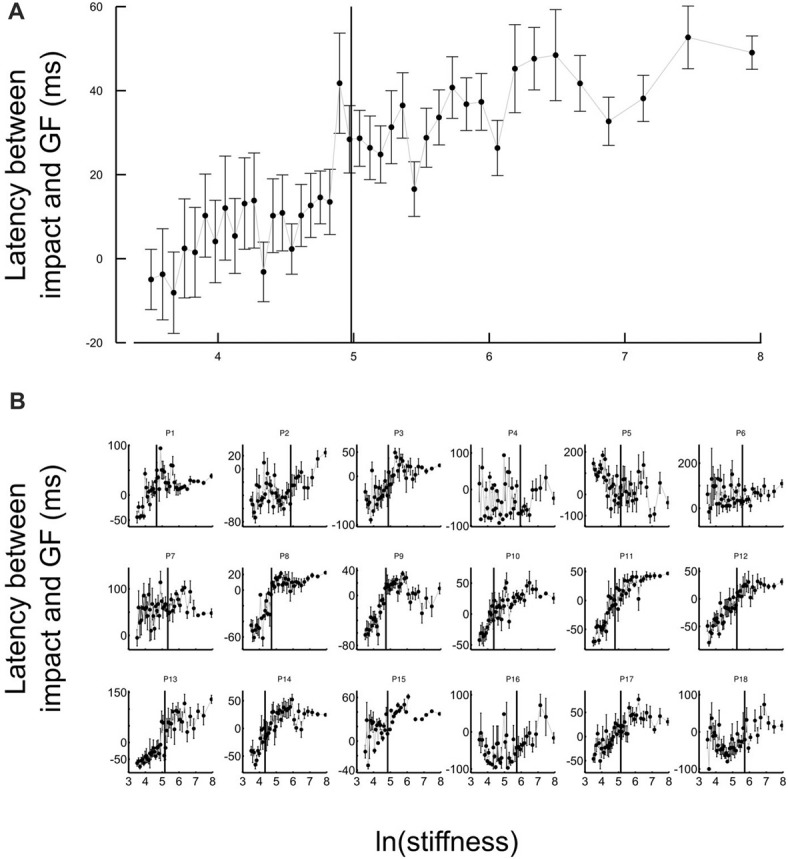
Grip force control switches for certain stiffness values. **(A)** Average lags across participants between grip force peak and impact as a function of the natural logarithm of stiffness. The same lags are plotted for individual participants in the lower panel **(B)**. The lags vary with stiffness. Positive values of latencies mean that grip force peaks lag impacts. Black cursors are positioned at the ln(stiffness) identified from grip force peaks that correspond to the average between the “Ascending” and “Descending” conditions. Error bars are SEM.

Another strategic change in grip force control is illustrated in Figure 4. The difference between times of grip force peaks and times of elastic force peaks is depicted as a function of the natural logarithm of stiffness (no significant difference between block conditions, *t*_17_ = 0.04, *p* = 0.973), for all participants together (Figure 4A) or individually (Figure 4B). The individual plots in Figure 4B reveal that most of the participants had a prominent transition in the dependency of the time difference as a function of natural logarithm of stiffness around a threshold value. Indeed, the delay seems to linearly increase from negative (leading latencies) and then plateau to a positive value (lagging time).

To reliably quantify this effect, we first extracted slopes of the linear fits of the lag in function of ln(stiffness) on individual participant data. Note that there was large inter participant variability in the quality of the change. Therefore, our analysis focused on determining the statistical significance of the difference in the slopes rather than on the analysis of the average behavior in each stiffness range. We partitioned the data in a low-stiffness and high-stiffness subset, according to the individual thresholds (averaged between the two block conditions) estimated as the value ln(stiffness) for which peak grip force occurred (see Figure 3B). We then statistically tested whether slopes differed between both stiffness conditions. To do so, we defined a random variable as the absolute difference of slopes between low and high stiffness conditions and bootstrapped that statistics (sample = 18, repetitions = 10,000, SD = 7.14). The CI was calculated by finding the interval containing 95% of the data (2.5 and 97.5 percentiles). As previously, we reasoned that if zero belonged to the 95% CI, then, the means could not be deemed as being different. In contrast, if zero is found outside the CI, then, slopes are different at *p* < 0.05. The results of our analysis show that slopes were different (*p* < 0.001) between low and high stiffness conditions (CI: 10.5–38.3). The Akaike information criteria (AIC) confirmed that a piecewise linear model describes our experimental data better than a single linear regression, despite the larger number of free parameters in the former. Indeed, the AIC for the piecewise regression is smaller than the AIC for the single regression both in “Ascending” (222.8 < 235.5) and “Descending” conditions (219.8 < 272.41).

**Figure 5:**
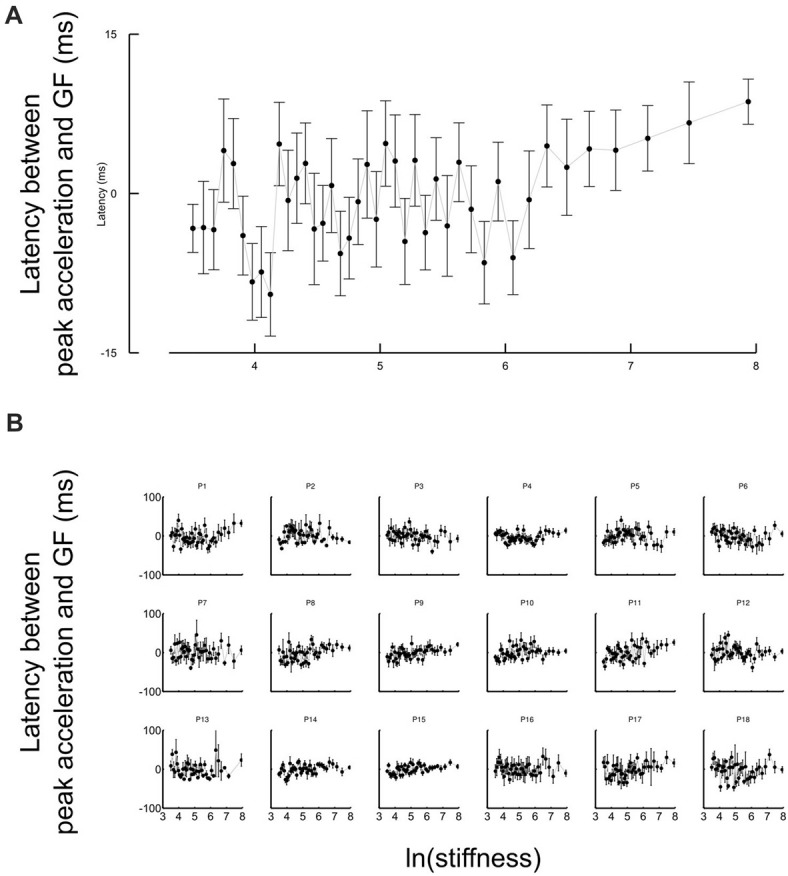
Grip force is synchronized with the inertial, acceleration-dependent, force whatever the stiffness values. **(A)** Average lags across participants between the first local grip force peak and the acceleration shortly after movement onset as a function of the natural logarithm of stiffness. The same lags are plotted for individual participants in the lower panel **(B)**. The lags are distributed around zero. Error bars are SEM.

In agreement with previous studies, grip force exhibited a first local peak that coincided with a peak of acceleration that occurred early after movement onset. In contrast to the observation of the change in the lag of synchronization of grip force with load force for the elastic load force, Figure 5A shows that the delay of the first, inertial, peak varied around zero on average for all participants and held true without exception on a participant basis (Figure 5B). The ANOVA confirmed this observation and failed to report any effect on the lag between these two peaks (all *F* < 0.2, all *p* > 0.551). A *t*-test revealed its value was not different from zero (mean = −2.3 ms; *t*_34_ = 0.3, *p* = 0.365). This highlights that the switching strategy was specific to the interaction with the elastic load force and the impact that characterized this interaction rather than a general change in lag between grip force and load force.

**Figure 6:**
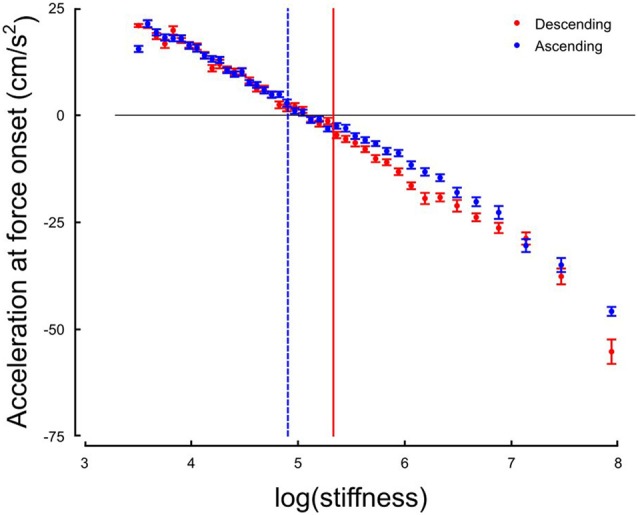
Hand acceleration at force onset (or expected) in function of natural logarithm of stiffness for Ascending (blue) and Descending trials. The horizontal black line is positioned at sign(acc) = 0. The two vertical cursors are positioned at the thresholds identified on the distribution of grip force peaks.

The system responds by switching the grip force lag to a different control strategy following a monotonic change in the environment. We hypothesize that this switch is triggered by a change in the value of a discrete variable that indicates the need for a qualitative change in the behavior of the system. One immediate candidate for such variable is the crossing of a stiffness threshold. Indeed, the switching in both increasing and decreasing series occurs around *k* = 147 N/m. However, crossing a stiffness threshold is not the only possible switching variable. In previous investigations, acceleration was shown to be a key information to perform tasks involving, for instance, eye-hand coordination (Binsted and Elliott, 1999; Helsen et al., 2000; White et al., 2012). Interestingly, we found that the stiffness threshold that we identified previously marked the transition between positive and negative accelerations at the time of force onset, that is, whether the hand of the participant was accelerating or decelerating when forces started acting on the hand (Figure 6). We identified, for each participant, the switch in the acceleration sign at impact, and plotted the switch in strategy as a function of the switch in the sign of acceleration at impact. The correlation was statistically significant across participants (*r* = 0.36, *p* = 0.030). This highlights that a correlation exists at a participant-level between these two variables, which however, does not imply causation.

## Discussion

We set out to understand the phase transition between two distinct grip force control strategies during tool-mediated interaction with elastic force fields. Participants interacted with springs with increasing or decreasing stiffness between two values and controlled grip force according to the expected dynamics in all trials, including in zero-stiffness catch trials. Peak grip force reached a maximum for an average stiffness of 147 N/m. Participants exhibited different qualitative behaviors related to the lag between peak grip force and elastic force in function of stiffness. For most of them, the lag increased with stiffness upon a certain threshold. Based on these observations, we suggest that the central nervous system acts as a hybrid controller that is characterized by continuous and discrete states and operates a phase transition upon a specific stiffness value, potentially triggered by the stiffness value, the sign of the acceleration at the time of the initial contact with the elastic force field, or other candidate switching variables.

### The Brain Modulates the Grip Force Lag to Optimize Stability

Some tasks are quintessentially complex, nonlinear and high dimensional, leading to postural instabilities and task uncertainties such as when we make contacts between two objects. Our results support a view according to which the central nervous system switches strategy in grip force control in the face of locally unstable tasks. Participants unconsciously modulate the lag between peak grip force and (expected, in catch trials) peak elastic load force. Stiff trials produce larger uncontrollable transitory forces (see Figure 6 in White et al., 2011). Our data is in line with the idea that long latencies are better suited to trials for which instability is largest. Indeed, as we showed previously, long latencies allow the viscoelastic properties of the skin to dissipate more energy than short latencies, for which the hand is stiffer (White et al., 2011). In other words, the latency is proportional to instability. Consistently, it was suggested that increasing the delay in a control loop may, in some cases and for certain values, improve stability (Malakhovski and Mirkin, 2006). Consequently, in stiff trials, grip force is smaller at the time when perturbations are maximal than a few tens of millisecond after. In addition to the ability to dissipate energy, lowering the forces has two other positive effects. First, the perturbing forces attributable to signal-dependent noise also decrease with lower forces (Jones et al., 2002; Hamilton et al., 2004). Second, excessive co-activation is energy greedy (Foley and Meyer, 1993; Sih and Stuhmiller, 2003). These grip force adjustment differences are happening within the range of grip forces that protect the participant from object slippage, as evidenced by the fact that none of the participants ever lost grip of the object.

This latency was not constant. Prior studies observed this latency without attempting to experimentally control it (Johansson and Westling, 1988; Johansson et al., 1992; Serrien et al., 1999; Turrell et al., 1999; Bleyenheuft et al., 2009), and found values consistent with the maximal latency (75 ms) we observed in stiff interactions between a hand-held object and a surface (White et al., 2011). In a recent study, we failed to alter that latency by changing stiffness of a virtual surface and direction of movement (White et al., 2011). This was likely the case because the stiff and soft targets were implemented with 1200 N/m and 240 N/m virtual springs, which were both above the stiffness values encountered here. However, in a different study, we observed modulation of latency during profound gravitational changes induced by parabolic flights that challenged participants by confronting them with fundamental environmental uncertainties (White et al., 2012).

### Switching Is Stiffness-Dependent

Perhaps the most striking observation is that the central nervous system switched between grip force strategies around a certain individual threshold identified through three independent observations. First, it marked the average stiffness at which grip force peaked (Figure 3B). Second, the piecewise linear fit had a remarkable point close to this stiffness (Figure 4A). Third, hand acceleration at force onset reversed its sign around that threshold. It is also worth reporting that a few hundreds of milliseconds before impact, in the very same trial, grip force exhibited a local peak that was synchronized with the small yet significant load force maximum due to inertia (Figures 5A,B). When comparing Figures 4A,B, 5A,B, it is very clear that participants predictively control grip force very differently when they are confronted to inertia or impacts. A more subtler adjustments also hold for low and high stiffness interactions.

Interestingly, while the lag was not statistically different between the two block conditions, there were two well identified global maxima in the peaks of the grip force as a function of stiffness. There is a hysteresis in the stiffness level at which the grip force is maximal: the maximum in the grip force appears at a higher level of stiffness in the ascending series than in the descending series. Such hysteresis behavior does not appear in the lag between grip force and load force. This suggests that during repeated interactions with the elastic force fields, the motor system identifies crossing a stiffness threshold, and switches the feedforward control strategy. Then, once evidence for having crossed the threshold is available and the success of the change in the strategy accumulates, the system reacts with a decrease of the magnitude of the grip force peak. The presence of a hysteresis is a signature of some inertia in the mechanisms that drive the switching.

The switching in grip force control strategy might be coupled with another example of a switch between two dichotomies in interaction with elastic force fields: during tool-mediated interaction with elastic objects, the motor system can choose between controlling movement trajectories to controlling interaction forces. Previous studies suggested that stiffness (Chib et al., 2006; Mugge et al., 2009) and stiffness discontinuity crossing (Nisky et al., 2008) lead to different weighting of position and force control in manual interaction. When participants interact with low-stiffness force fields, they control kinematics, and estimate the stiffness of the elastic field based on integration of position information with sensed forces. With increasing stiffness, the reliability of stiffness estimation deteriorates in accordance with Weber’s law (Jones and Hunter, 1990). When participants interact with elastic force fields with higher stiffness (Chib et al., 2006; Mugge et al., 2009), or more frequently cross stiffness discontinuity (Nisky et al., 2008), they favor control of interaction forces rather than the control of kinematics. When this transition happens, the central nervous system might start estimating the compliance of the elastic field (the ratio between the displacement and the force that caused it) rather than its stiffness (the ratio between the force and the displacement that caused it), resulting again in reliable estimates. Such view of different estimation is consistent with our observation that peak grip forces are largest around the transition and are smaller for very high or very low stiffness levels. It is also strikingly consistent with the threshold of around 100–200 N/m in the stiffness-dependent weighting of force and position feedback (Mugge et al., 2009).

If indeed a stiffness threshold is used as a switching variable, to use this information in control of robotic interfaces, it is important to model how the brain estimates stiffness. Various computational models were proposed, including: peak force divided by perceived penetration (Pressman et al., 2007, 2008, 2011), or regression of force over position or position over force data (Nisky et al., 2008, 2010, 2011). Another proposed measure is Extended Rate Hardness, a measure of the perceived hardness of a surface based on rate of force change and penetration velocity (Han and Choi, 2010). Skin deformation accompanying the probing also likely plays a role in perception of stiffness (Quek et al., 2014, 2015; Farajian et al., 2017). Here, we do not attempt to spill more light on this matter, but it is likely that the estimated stiffness is used in the process of the switching.

We investigated the behavioral aspects of the switching and its potential underlying switching variable. The question remains open which neural structures operate this switching. In previous studies, we have shown that the left supplementary motor area (SMA) is a crucial node in the network that processes the internal representation of object dynamics (White et al., 2013) leaving this neural structure as a potential candidate to host the decision variable that controls the phase transition. We also showed that the posterior parietal cortex (PPC) is involved in the perception of stiffness (Leib et al., 2016). Other candidate areas may include the cerebellum and the insula. Several studies reported bistable states of Purkinje cells in the cerebellum that may serve as a switching trigger (Yartsev et al., 2009). Finally, the insula seems to be involved in switching between the executive control and default networks (Sridharan et al., 2008).

Finally, it is worth pointing out that our results are apparently at odd with the fact participants cannot switch control policies in reaching movements between two opposing viscous force fields (Karniel and Mussa-Ivaldi, 2002). The occurrence of each force field was cued. The authors suggested that competition occurred between two different internal models that could not co-exist in the brain. In our experiment, switching occurred while the nature of the force field varied predictively as well. However, its variations were far more continuous than in the Karniel and Mussa-Ivaldi experiment. Therefore, we suggest that in our paradigm, the brain could rely on a single internal model and re-estimate the upcoming stiffness value based on recent history. As shown, the switched behavior took some trials to really occur after the thresholds were broken. Instead, in the Karniel and Mussa-Ivaldi study, the difference between force fields were more contrasted, making it difficult to re-estimate the force field based on a single parameter that took very different discrete values. We think that switching or learning is possible if the nature of the force field changes continuously and gently, whatever the complexity of the changes (for instance, we showed recently that participants could immediately adapt grip force in new gravitational phases generated by a centrifuge (Barbiero et al., 2017; White et al., 2018)). In addition, we previously found that the control of grip force may be characterized by different control policies than perception or manipulation—for example, during interaction with elastic force fields, delay causes bias in perception but not in the control of grip forces (Leib et al., 2015). Moreover, and perhaps closer to our current study, the predictive control during lifting of series of objects with increasing mass (Mawase and Karniel, 2010) was fundamentally different from during reaching with perturbing force fields with a series of increasing viscosity parameters (Mawase and Karniel, 2012).

### Limitations and Perspectives

We should however also underline two limitations of the present study. First, we failed to explain these results within a fully coherent, average behavior. Instead, we found some idiosyncratic changes in strategy. This new question should be addressed in a follow-up experiment aiming at identifying what caused these switches. Second, our data exhibit large variability, which is inevitable when studying the interaction between mechanical interactions and physiological processes. Future investigations should improve the technical design of these experiments. Our contribution paves the way toward using switched systems theory in modeling human motor control and opens new research questions as to the nature of the discrete state variables that drive the switching between different control strategies.

How can these results be employed in the control of robotic systems? Identifying human control strategies in object manipulation is crucial for developing efficient control algorithms for a variety of human-operated robotic applications ranging from tele-operated surgical robotics to smart prostheses. Modulation between grip force and load force in human grasping allows for securing held objects against slippage without applying excessive forces. This modulation is impossible in the absence of force feedback (Gibo et al., 2014). Therefore, in state of the art tele-operation robot-assisted surgery systems, users apply unnatural grip force control strategies (Gibo et al., 2014), likely leading to suboptimal performance. Adding some form of force feedback about the load force of manipulated objects contributes to natural coordination between grip force and load force. Our current results suggest that the force feedback that is presented to the user should be designed in a manner that the switching strategy can be employed. If this is impossible due to limited tele-operation control gains, the tele-operated gripper can incorporate local smart switching in grip force control. Similar ideas may be implemented in next generation prostheses to facilitate natural manipulation of objects. Future studies are needed to develop such human-inspired controllers and test their potential benefits compared to state of the art grippers and prostheses controllers.

To conclude, we show here evidence that the central nervous system adopts qualitatively different grip force controls to cope with impact-like environments. Our results show that the central nervous system acts as a switching system. Our findings may have very practical implications since human-machine interfaces nowadays involve haptic feedback, but many applications of fine object manipulation are missing haptic feedback, such as robot-assisted surgery and prosthetics.

## Author Contributions

OW and AK designed the experiments, collected and analyzed data. OW, AK and IN interpreted the results. MB collected data. OW, AK, CP, MB and IN contributed to writing the manuscript, manuscript revision, read and approved the submitted version.

## Conflict of Interest Statement

The authors declare that the research was conducted in the absence of any commercial or financial relationships that could be construed as a potential conflict of interest.
